# Postpartum people’s experiences of and responses to the COVID-19 pandemic during the first year of the pandemic: A descriptive qualitative study

**DOI:** 10.1177/17455057231157480

**Published:** 2023-02-27

**Authors:** Hamideh Bayrampour, Ming Yee Emily Tsui

**Affiliations:** Department of Family Practice, Faculty of Medicine, University of British Columbia, Vancouver, BC, Canada

**Keywords:** coronavirus disease 2019 pandemic, health services, perinatal period, qualitative descriptive study

## Abstract

**Background:**

Most evidence on the impact of pandemic on perinatal population’s experiences has reported such effects in a portion of the pandemic.

**Objectives:**

The aim of this study was to understand the postpartum people’s experiences of and responses to the coronavirus disease 2019 pandemic during the first year and to identify their health care needs.

**Design:**

This is a descriptive qualitative study.

**Methods:**

This study was conducted in British Columbia, Canada, between March 2020 and April 2021. Participants (N = 268) were at 4 months postpartum and were recruited as part of the Pregnancy Specific Anxiety Tool study through prenatal care clinics and classes, community laboratory services and social media. Qualitative data were obtained using six online open-ended questions and were analysed using thematic analysis.

**Results:**

Findings were grouped under five central themes: protecting baby (with three categories including hypervigilance, constant decision-making to find balance and developmental issues); psychological adjustments (with three categories including coping, anxiety and grief); experience of isolation and lack/loss of support (with two categories including isolation and loss of expected support); unexpected interruptions and life events (with four categories including interrupted maternity leave, unexpected changes/life events, positive impacts and interruption in health care services); and perceived postpartum care needs (with five categories including in-person visits, allowing support persons, providing information/education/support groups, mental health and social support and pro-active check-ins).

**Conclusion:**

Several impacts of the pandemic persisted throughout the first year, particularly isolation and lack of support. These findings can inform responsive health care services to address the emerging needs of postpartum people throughout the pandemic.

## Introduction

Coronavirus disease 2019 (COVID-19) has greatly impacted population health across the globe and has been associated with increased mortality and morbidity.^1^ Emerging evidence shows an increase in overall adverse perinatal outcomes^2–4^ and postpartum events compared with the non-COVID-19 period.^5^ The COVID-19 pandemic has also had a substantial impact on pregnant and postpartum persons’ psychosocial wellbeing.^2,6–8^ In a sample of 603 postpartum participants from Ontario, Canada, Layton et al.^9^ found that compared to the pre-pandemic cohort (March 2019 to March 2020), the pandemic cohort (April to October 2020) had 65% and 46% higher odds of experiencing postpartum depression and anxiety, respectively. In a population-based analysis in Ontario, Canada, Vigod et al. also reported elevated physician visit rates for mental health disorders (e.g. depression, anxiety, substance use disorders) in the postpartum period between April and November 2020.^10^

The COVID-19 pandemic has imposed significant challenges on various sectors of health care system. In response to the pandemic, maternity care services similar to other areas of health care were quickly altered to ensure a safe provision of care in the midst of the pandemic. These rapid system alterations have had substantial and potentially prolonged impact on the maternity care experience of both providers and patients. A qualitative synthesis of 48 studies showed that regardless of the studies’ settings, the alteration in maternity care structure, process, provision and delivery in various parts of the globe often induced mixed emotions, prominently negative emotions, for both women and maternity care providers.^11^ This review also highlighted that the pandemic has intensified the extant inequalities and disparities in perinatal services.^11^ A qualitative study of 57 postpartum people in Ontario, Canada, conducted between June 2020 and January 2021 showed an exacerbation of pre-existent challenges among people with underlying mental health conditions or complicated pregnancies. The authors noted that it is possible that the pandemic-imposed restrictions such as absence or limited number of support persons may have intensified the pre-existent psychosocial and health disparities during the postpartum period.^12^ The accumulating evidence indicates that the inappropriate adaptation of health care services in response to the pandemic may have caused inconsistencies in care, cancelled or delayed appointments, conflicting recommendations and unmet health care expectations and needs and may have created uncertainty and anxiety for perinatal people.^11^

To design and implement responsive maternity care services during public health crises such as pandemics, it is essential to understand the impact of pandemic and its restrictions and subsequent alterations in the provision of care on the lived experiences of childbearing people and to identify their health care needs. The objectives of this study were (1) to understand the postpartum people’s experiences of and (2) responses to the COVID-19 pandemic during the first year of the pandemic, from March 2020 to April 2021, in British Columbia (BC), Canada, and (3) to identify their health care needs. Most current evidence on the impact of the pandemic on the postpartum people has reported such experiences in a portion of the pandemic. This study will provide an insight throughout the first year of the pandemic.

## Methods

### Study design and setting

We used a qualitative descriptive framework, an approach relevant for understanding health care inquiries and interpreting participant input in everyday words.^13^ We applied this approach as the purpose of this study was to understand and describe our participants’ experiences of and responses to the COVID-19 pandemic. We used the consolidated criteria for reporting qualitative research (COREQ) checklist to report findings.^14^ This study was conducted in BC. The first community transmission of COVID-19 (i.e. transmission source was unknown) was reported on 5 March 2020 in BC. Following the announcement of a state of emergency on 18 March by the government of BC, the province entered a 2-month stay-at-home closure period. The weekly tally of cases peaked at the end of March 2020 (first wave). After re-opening of services in June, the weekly incidence of COVID-19 increased in mid-August. Weekly cases spiked in mid-October with a steady sharp increase throughout November that persisted until December 2020 (second wave). Weekly incidence declined in January and February 2021 followed by another steady increase in March and a spike in April, reaching the highest recorded weekly incidence since the start of the pandemic (third wave). With widespread public vaccinations from May 2021, the number of new cases declined substantially and stayed low for the spring and early summer 2021.^15^ When the pandemic emerged in BC in March 2020, our team was conducting phase 3 of the Pregnancy Specific Anxiety Tool (PSAT) study^16^ with the aim of developing a screening tool for pregnancy anxiety (July 2019 to April 2021). Pregnancy data collection for the PSAT study occurred between July 2019 and May 2020. Four-month postpartum data were collected between December 2019 and April 2021. This study is based on the 4-month postpartum data collected between 20 March 2020 and 19 April 2021. Pregnancy data related to the COVID-19 pandemic (20 March to 31 May 2020) have been reported previously.^17^

### Participants

Participants’ recruitment occurred through prenatal care clinics and classes and LifeLabs (i.e. blood and other specimen collection laboratories) across BC as well as social media (i.e. Facebook page and groups, Twitter, Instagram). Individuals were eligible to participate if they were pregnant (at any gestational age); resident of BC; and able to read, write and speak in English. Being 18 years old and younger was an exclusion criterion. Participants were followed up until 4 months postpartum.

### Data sources and collection

The purpose of the original PSAT study was to develop a screening tool for pregnancy-specific anxiety. Hence, the study included several measures of mental health. When the pandemic emerged in BC in March 2020, we were in the midst of data collection for the PSAT study. To understand the potential impact of the pandemic on our participants, our team developed six open-ended questions. Some questions were inspired by the findings of a previous study on the influenza A pandemic (H1N1)^18^ that helped us to identify the broader areas of focus for some questions (e.g. perceptions and coping). All questions were worded and constructed by the first author (H.B.) and the PSAT team. The questions were open-ended and attempted to capture the experiences of the participants during the pandemic (Table 2). The questions were administered both during pregnancy and the postpartum period. The pregnancy and postpartum questions were similar, and they asked participants about their thoughts and feelings about the pandemic, its impact on their life, their physical and mental health and on the baby, how they cope and how their health care provider could support them during the pandemic. To accommodate an immediate data collection purpose (i.e. as of 20 March 2020), the questions were not piloted. Data were collected through REDCap, a Canadian-based secure web application to collect research data. Almost no word limit was imposed on the response fields (2000 words). The default visible text box size for each answer field would fit 35–40 words but it was expandable. Basic demographic data were retrieved from the pregnancy survey.

### Data analysis

Thematic analysis, a systematic approach ‘to find repeated patterns of meaning’,^19^ was used to analyse the data.^19–21^ Two female analysts (H.B., an assistant professor with a background in midwifery and research interests in perinatal mental health and pregnancy outcomes and experience in conducting qualitative and mixed methods studies, and E.T., a research assistant with a Bachelor of Science degree in biology and psychology and experience in working as a crisis counsellor and prenatal genetic counselling assistant) read participants’ input to immerse in the data and become familiar with the content. Then, the two analysts coded participants’ input independently and compiled data relevant to each code to create the initial codes. For the first 15 participant codes, H.B. received feedback from two colleagues. Once the initial codes were generated from all data, H.B. and E.T. searched for potential themes in collated data by sorting the similar codes to create an overarching theme. Through an iterative process, H.B. and E.T. reviewed the candidate themes for the coherency of each individual theme as well as for precise representation of the data by all themes and how they fit together. Once an initial refined set of themes was developed, we further examined the essence of each theme to refine and define the theme and how all themes were related to one another. At this stage, we also considered assigning more expressive names to each theme to quickly convey the underlying meaning to a reader. For example, we replaced the term ‘being extra cautious’ with ‘hypervigilance’. Microsoft Excel was used to manage the data.

Rigour (analytic validity and reliability)^22,23^ was established by an independent data analysis by the two analysts, verbatim quotes and describing participants’ characteristics and contextual information to enable assessment of applicability of findings to other settings and populations. Our findings included identification of contrary cases. For example, our findings showed that some participants experienced and perceived positive impacts during the pandemic, in contrast to other participants who had negative experiences. Another example is the great appreciation and acknowledgement of health care provider support reported by several participants, while some participants noted negative feedback. We reported these contrary cases that attest to credibility of the findings of this study.

## Results

Two hundred sixty-eight participants provided data for this study. The mean age of participants was 31.75 (4.14) years. Majority of participants were multiparous (60%) and gave birth to a singleton pregnancy (99%). The mean gestation age at birth (weeks) and birth weight (grams) were 39.15 (1.39) and 3478 (484), respectively. Most participants (99.6%; n = 267) identified their gender as female. Approximately, 70% of the participants resided in Vancouver Coastal or Fraser Health Authorities (Table 1). All participants answered the open-ended question 5 (N = 268). A total of 5, 2, 3, 7 and 30 participants did not answer questions 1, 2, 3, 4 and 6, respectively (Table 2).

**Table 1.** Characteristics of participants (N = 268).

Variables	
Basic characteristics	
Age (years), mean (SD)	31.75 (4.14)
Born in Canada, n (%)	206 (76.9)
Racial or ethnic background^a^, n (%)	
White	194 (72.4)
East/South Asian	49 (18.3)
Indigenous	6 (2.2)
Other	18 (6.7)
Married/Common-law or live-in partner, n (%)	264 (98.9)
Education: university degree, n (%)	197 (73.5)
Household income^b^, n (%)	
<US$40,000	14 (5.2)
US$40,000–US$99,999	104 (38.8)
⩾US$100,000	147 (54.9)
Gender, n (%)	
Female	267 (99.6)
Male	0
Non-binary	1 (0.4)
Geographic region, n (%)	
Fraser Health	94 (35.1)
Vancouver Coastal Health	92 (34.3)
Interior Health	37 (13.8)
Island Health	34 (12.7)
Northern Health	11 (4.1)
Pregnancy characteristics	
Primigravida, n (%)	106 (39.6)
Problems or complications during pregnancy, n (%)	83 (31.0)
Caesarean birth	85 (31.7)
Newborn/Infant characteristics	
Gestational age at birth (weeks), mean (SD)	39.15 (1.39)
Birth weight (grams), mean (SD)	3478.75 (483.83)
Infant received all routine vaccines for age^c^, n (%)	247 (92.2)

a Missing data for one participant (0.4% of the sample population).

b Missing data for three participants (1.1% of the sample population).

c Participants completed the postpartum survey between 3 months 0 days and 4 months 30 days postpartum.

**Table 2.** Open-ended survey questions and the number of participants who provided a response for each question (N = 268).

Question	Number of participants who provided a response
1. As a parent, what are your thoughts and feelings about the recent pandemic of coronavirus (COVID-19)?	263
2. How has this outbreak impacted your life and your plans if any?	266
3. What are your thoughts on whether this pandemic has impacted your baby?	265
4. What are your thoughts on whether this pandemic has impacted your physical and mental wellbeing?	261
5. How do you cope with this pandemic?	268
6. How can your health care provider(s) support you during this pandemic?	238

COVID-19: coronavirus disease 2019.

**Figure 1.**
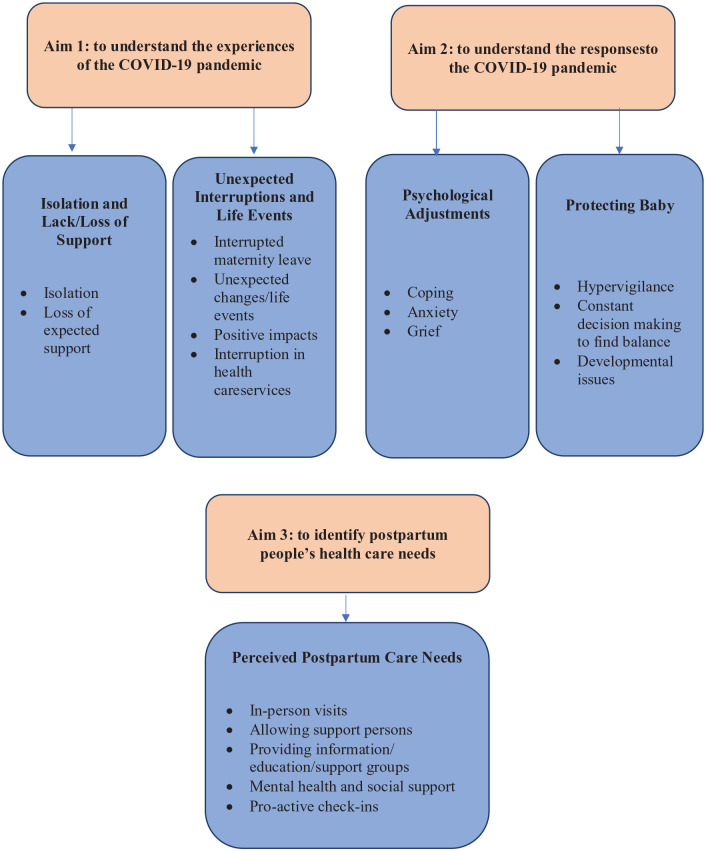
Study aims, themes and sub-themes. COVID-19: coronavirus disease 2019.

Findings were grouped under five central themes (Figure 1):

a. Protecting baby with three categories including hypervigilance, constant decision-making to find balance and developmental issues;
b. Psychological adjustments with three categories including coping, anxiety and grief;
c. Experience of isolation and lack/loss of support with two categories including isolation and loss of expected support;
d. Unexpected interruptions and life events with four categories including interrupted maternity leave, unexpected changes/life events, positive impacts and interruption in health care services;
e. Perceived postpartum care needs with five categories including in-person visits, allowing support persons, providing information/education/support groups, mental health and social support and pro-active check-ins.

Table 3 illustrates themes and sub-themes and representative quotes.

**Table 3.** Themes and sub-themes developed from qualitative data and representative quotes, March 2020 to April 2021, N = 268, British Columbia, Canada.

Theme/Category	Representative quote
Protecting baby	
Hypervigilance	Quote 1: ‘I feel as though our lives have been upended and my biggest priority is keeping our baby safe and also keeping ourselves healthy because I don’t know how we would be able to care for her if one of us got sick (this gives me a great deal of stress).’ P11; 11 April 2020 Quote 2: ‘If we didn’t have a newborn, I would be less cautious with the pandemic and anxious. But because our baby is so precious and vulnerable, we take strict precautions. No one has held our child but myself and my partner. I find it sad that he doesn’t have interaction with more people.’ P79; 10 July 2020
Constant decision-making to find balance	Quote 3: ‘I’m stressed. I’m trying to find the balance of protecting myself and my baby from any potential exposure while also trying to keep sane by still going out. It’s a hard adjustment.’ P42; 3 June 2020 Quote 4: ‘I feel guilty asking people to wash their hands or wear a mask or to stay away.’ P183; 19 November 2020 Quote 5: ‘It’s has been challenging trying to make the right/safe decisions for my kids ( do I still send my two year old to daycare? Is it safe to take my kids to the grocery store with me?) and hard not having as much in person family support due to travel and visiting restrictions.’ P198; 7 December 2020
Developmental issues	Quote 6: ‘I suspect that his immune system is lacking from being so sheltered.’ P184; 19 November 2020 Quote 7: ‘I wonder if it will cause an increase in separation anxiety once others can hold her’ P38; 29 May 2020 Quote 8: ‘I am terrified of the possible developmental impact this pandemic could have and am not happy we get to be the unknowing test subjects of finding out the impact of limited face to face exposure of a newborn to a variety of faces. I am aware of how rapidly babies develop and learn. And I am aware that their facial recognition is generally set by about a year, so how will 90+% of the humans they see being masked impact her develop? It can’t be normal.’ P228; 5 January 2021 Quote 9: ‘I was not a fan of screen time but with this pandemic we are FaceTiming almost daily and because I spend so much time at home she gets some extra screen time since I watch more tv than if I was able to be out visiting people and doing activities.’ P228; 5 January 2021
Psychological adjustments	
Coping	Quote 10: ‘I drink more than I should at the end of the day if it’s been really hard.’ P30; 15 May 2020 Quote 11: ‘Online zoom mom groups, weekly phone with family/friends, weekend distancing outdoor visits with family, home workouts, husband works from home, podcasts, daily family walks, pets, trade baby off to get some alone time.’ P86; 19 July 2020
Anxiety	Quote 12: ‘It is extremely anxiety-inducing for me. I worry about even taking my baby out for a walk and how close other people are getting to us. My partner has done all the shopping for our household.’ P11; 11 April 2020 Quote 13: ‘This pandemic has added to my anxiety as a new mom because I’m much more isolated, have limited help available to me, and limited access to medical and health care (i.e. physio and massage therapy for L + D recovery, having my son properly assessed for a tongue tie, breastfeeding support).’ P62; 26 June 2020 Quote 14: ‘I am more socially isolated, but going out in public is often a source of anxiety because of how fearfulness has altered human relations. People are can be very aggressive, rude, officious, and authoritative. This can be very difficult for me emotionally, because I don’t get out much and when I do, there are frequent negative interactions.’ P186; 20 November 2020 Quote 15: ‘I have covid anxiety, I avoid going to indoor public spaces or meet up with people indoors . . . Since babies cannot wear masks or get vaccinated until age 2, I worry if strangers on the street (outside) will sneeze/cough near my baby.’ P259; 12 March 2021
Grief	Quote 16: ‘Makes me feel disappointed and grieving over how I thought maternity leave would go.’ P40; 29 May 2020 Quote 17: ‘I feel I have lost some of the fun and enjoyment of new motherhood by not being able to share the experience with others due to COVID-19. We have not been able to enjoy sharing our baby with family and friends in the way we had imagined due to the COVID-19 restrictions and concerns.’ P53; 12 June 2020 Quote 18: ‘I feel fairly robbed of the experience of being a first time parent.’ P148; 28 September 2020 Quote 19: ‘It robbed us of so many things and experiences we can’t get back. I hate having to wear a mask in public so my baby cant see my smile or my mouth moving while I talk . . . Not having a baby shower or having support in the delivery room was really tough.’ P183; 19 November 2020 Quote 20: ‘Sense of loss. Missing out on activities and spending time with other new parents.’ P251; 13 February 2021
Experience of isolation and lack/loss of support	
Isolation	Quote 21: ‘I was wanting to get out and do exercise classes with baby, but mentally I feel like I am stuck inside with baby, and that makes me feel trapped.’ P111; 19 August 2020 Quote 22: ‘The pandemic has been extremely isolating and has limited my ability to function as an adult, because I did not take transit throughout my pregnancy and now only take the bus or cabs to get to medical appointments. Being socially isolated has made it harder to manage the anxiety and exhaustion that comes with early parenthood and sleep deprivation. It has also put a lot of pressure on my marriage, although we are still very loving and supportive. It is difficult to be in a tiny apartment together, with no one to come and help and nowhere for either one of us to go that isn’t outside. In the middle of winter, it was very difficult.’ P255; 26 February 2021
Loss of expected support	Quote 23: ‘Support has dwindled, I’m mostly alone with two kids. Mostly living in survival mode. We can’t plan for anything, it’s just day by day.’ P30; 15 May 2020 Quote 24: ‘My mother could not join my labour and delivery experience as she did with my first hospital visit. Our stay in maternity was lonely and isolating; my daughter experienced jaundice after birth which equaled a longer hospital stay and unplanned time away from our toddler.’ P221; 1 January 2021
Unexpected interruptions and life events	
Interrupted maternity leave	Quote 25: ‘Husband was laid off for a few months and went into a depressive slump. I started my maternity leave a few months early.’ P170; 26 October 2020 Quote 26: ‘Husband is having difficulty finding work. This may mean I will return to my job much sooner than I thought.’ P34; 24 May 2020
Unexpected changes/life events	Quote 27: ‘My husband and I moved in my with in-laws in Kamloops for two months as we did not feel Vancouver was the safest place to be with our newborn during the pandemic.’ P53; 12 June 2020
Positive impacts	Quote 28: ‘I think it’s had a positive impact. My husband is home all the time whereas he would have been gone most days. We also aren’t traveling much and disrupting our son’s daily routine. His life is incredibly calm and happy’ P25; 12 May 2020
Interruption in health care services	Quote 29: ‘I’m off my midwife care and my family GP quit her practice. I wish we had a provider for her wellness visits. I get anxious thinking about not having a doctor for her.’ P36; 28 May 2020 Quote 30: ‘We didn’t get our last two appts with our midwife, we haven’t seen any care provider since our 2 week checkup. Immunizations were very hard to get, and scary because if she had a reaction what happened if we needed to go to the hospital.’ P30; 15 May 2020 Quote 31: ‘Right away I can see that my baby is getting very different medical attention. He will be meeting his family Doctor for the first time at 4 months old.’ P184; 19 November 2020 Quote 32: ‘Health issues are delayed to get care for as HCP are reluctant to see you in person. For first time ever have been diagnosed with PPA [ postpartum anxiety]. Even that has minimal “treatment” as it took over a month for reproductive mental health to call, and three months for an appointment.’ P265; 2 April 2021
Perceived postpartum care needs	
In-person visits	Quote 33: ‘I haven’t seen my health care provider since 4 weeks postpartum and what I really need right now is a physical. On the phone isn’t enough for a postpartum visit. My son also hasn’t been physically checked since 4 weeks of age. Just having peace of mind that he is good would help alot.’ P39; 29 May 2020 Quote 34: ‘I wish they wouldn’t rush our medical appointments. I so rarely get to see them, but the pre-visit telephone interview with shortened in-person assessments really feel rushed and I don’t feel nearly as connected.’ P94; 4 August 2020 Quote 35: ‘The phone calls are helpful but I need someone to look at my baby and tell me if she is growing ok and healthy and her head isn’t too messed up in shape.’ P136; 17 September 2020
Allowing support persons	Quote 36: ‘Allow both parents in the room for big appointments such as vaccinations, especially if one parent is having a panic attack.’ P67; 28 June 2020 Quote 37: ‘Couldn’t have family in the hospital or even see some of them after baby was born because it’s too high risk for travel.’ P197; 5 December 2020
Providing information/education/support groups	Quote 38: ‘Provide us with updated accurate information about the impact of the virus on babies and steps to take to stay safe. While still being realistic about the need for social support with a new baby.’ P48; 8 June 2020 Quote 39: ‘I’m often confused and torn about how to understand and interpret the guideline and orders because they seem to change so often and rarely have actual consequences to them. I am frustrated that there are no clear guidelines on pregnancy and newborn health relating to Covid and that some of these recommendations could be causing more harm than good for little ones.’ P228; 5 January 2021
Mental health and social support	Quote 40: ‘I think there needs to be a restructuring of perinatal mental health in general, and especially at times when there are additional stressors, such as a global pandemic. I have been asked by a few different healthcare providers “how are you doing?” While this is nice, in my opinion it doesn’t really allow for open and honest answers given that this question comes up at the same time as checking on physical recovery from birth or at well baby appointments when the focus is on other things. I don’t know what the solution is, but perhaps a question like “what are your challenges” in an appointment that is just focused on mental health may be helpful. That said, I also recognise that the healthcare system is overtaxed and I am lucky to have great healthcare providers who are doing the best they can in a foreign situation.’ P46; 4 June 2020 Quote 41: ‘Being available. Listening thoroughly. Understanding that there is a baseline anxiety with COVID, on-top of the baseline anxiety of being a new parent.’ P62; 26 June 2020 Quote 42: ‘Healthcare providers are some of the only people I was able to interact with during pregnancy and postpartum! Having healthcare providers who take the time to check in with me on more than just the primary physical treatment needs has been beneficial for me. I’ve also appreciated it when my doctor is able to consolidate needs into one appointment (like checking my baby, giving a vaccine, and checking in on my health needs) so that we don’t have to enter health care settings more frequently.’ P264; 30 March 2021
Pro-active check-ins	Quote 43: ‘It would have been nice for them to actively reach out more. So far, most appointments are now over the phone unless being there in person is necessary. Almost makes you feel “bad” for phoning in with questions etc.’ P83; 17 July 2020

### Protecting baby

The main response and the central goal for many participants during the pandemic were to protect baby. However, due to pandemic-imposed challenges, participants often felt overwhelmed and anxious about how to best safeguard their infants.

#### Hypervigilance

Many participants reported the feeling of being more cautious with regard to pandemic safety measures than other people in their lives (quotes 1 and 2 in Table 3). Several participants reported ‘watching everyone’ and not trusting anyone with their baby. A participant described this attitude as ‘hypervigilance’ and several others as ‘paranoid’. One participant also reported coming up with a script to set boundaries with people who wanted to approach baby:

The doctor says/recommends nobody sees or holds baby before her 2 months shots but if we decide to let anyone meet her they need to ______ [do this], [do this] ________, and [this] _____. If you can’t follow that then you can see baby through FaceTime and pictures.

A participant described ‘carrying the mental load of extra protection’ as taxing, which created substantial anxiety. Taking safety precautions sometimes puts strains on participants’ relationships with their partner and extended family, contributing to their poor mental health and diminished instrumental support.

#### Constant decision-making to find balance

Participants described constant struggles to make daily decisions and find balance between protecting baby and not interfering with child’s social development. Decisions around family visits and sending other children to school/daycare were particularly highlighted (quotes 3–5). Despite taking several precautions, some participants questioned their decisions and wondered whether masks and social distancing were right choices or whether these measures could harm baby’s development over time. Some participants reported feeling ‘guilty’ for making any choices or the feeling of ‘standing between baby and grandparents’. A first-time mother noted that ‘I feel like it’s me keeping my baby from them and not COVID’.

#### Developmental issues

Concerns about physical, mental and social development of the infant were raised by several participants (quote 6). Physical development concerns were mainly focused on how an excessive use of sanitizing products would affect the child’s microbiome and immune system in long term. Others also expressed concerns that a lack of social interactions could contribute to lack of exposure to common bacteria or viruses beneficial in building a robust immune system. Mental and social development concerns were focused on overall social development and particularly the risk of developing social and separation anxiety due to limited/lack of interactions with other people and infants (quote 7). As the pandemic continued and wearing masks became a norm, some participants also expressed concerns about the development of facial emotion recognition abilities due to face coverings (quote 8). Increased screen time during pandemic and its developmental effect were other areas of concern (quote 9). Some participants were also worried that their own anxiety and stress both during pregnancy and the postpartum period may have had negative impacts on baby.

### Psychological adjustments

#### Coping

Many participants reported taking it ‘one day at a time’ to deal with psychological strains of the pandemic. Avoiding news and limiting media exposure, talking to partner, doing meditation, being outdoors, virtual calls with family/friends and staying positive were reported frequently (quote 11). Lack/limited access to coping mechanisms such as working out and being with support circle particularly during the first wave of the pandemic were frequently reported to contribute to poor mental health. Some participants also reported drinking alcohol to deal with multiple stresses (quote 10).

#### Anxiety

Anxiety was frequently reported, particularly during the first wave of the pandemic. As the pandemic continued, some participants reported a decline in their initial anxious feelings. Anxieties were focused on unknowns, how long the pandemic is going to last, becoming ill and being separated from baby/children or not being able to take care of them, having limited support and access to health care services and breastfeeding support, how to keep baby safe outside home (e.g. how close others getting to baby or sneeze/cough near baby) and concerns about baby contracting the infection (quotes 12–15). Some participants reported concerns about COVID vaccine being mandatory. A first-time mother noted that she is worried that she will have to choose between receiving vaccine and breastfeeding. Others reported concerns about administering COVID-19 vaccine to their infants.

#### Grief

The feelings of ‘being robbed’ of the normal experience of motherhood and maternity leave were frequently reported (quotes 16–19). Some participants noted that ‘the joy of having a new baby has been taken away’ from them. Grief and disappointments about losing opportunities to take part in parent/baby groups and connect with other new parents were recurring themes. Many participants also reported grieving about their children ‘missing out a normal childhood’ and their families missing out on being part of their infants’ life (quote 20).

### Experience of isolation and lack/loss of support

#### Isolation

The central experience throughout the pandemic was ‘isolation’. Some participants stated that the postpartum period is already an isolating and challenging experience and that the addition of the pandemic has made this life stage lonelier. Feelings of being ‘stuck’ and ‘trapped’ were expressed frequently (quote 21). While some used online platforms to connect with others, many felt this was not sufficient. Not having family members’ visit contributed to the sense of isolation, especially for immigrants with families living overseas. Lack of social connections also decreased the instrumental support that postpartum people were hoping to receive. This was particularly challenging for mothers with generally low social support such as people with no partners or newcomers. Some participants noted that they refrained from seeking support due to the fears of virus contaminations or ‘being judged for being around others’. Several participants reported that the isolation, lack of support and uncertainties resulted in declining mental wellbeing or difficulty managing ongoing mental health challenges (quote 22).

#### Lack/loss of support

Closely related to isolation, loss of expected support developed as another substantial experience (quotes 23–24). Not having instrumental support from family and friends, unavailability of childcare and not having a break from parenting were reported frequently. Particularly, many participants noted that they were hoping to attend postpartum support groups to build connections with other mothers and receive peer support. Due to lack of access for young children’s activities, some multiparous participants also noted pressure to entertain and keep their other young children stimulated while taking care of the newborn.

### Unexpected interruptions and life events

#### Interrupted maternity leave

Some participants reported interruptions in maternity leave and an earlier return to work than initially planned. Partner’s unemployment was reported frequently as the main reason for short maternity leaves (quotes 25–26).

#### Unexpected changes/life events

Many participants experienced unexpected life events and changes caused by the pandemic. These unexpected changes contributed to psychological challenges and added a layer of anxiety. Some participants reported moving to different and less crowded regions to protect baby or moving in with their extended family due to financial strains and unemployment (quote 27).

#### Positive impacts

Several participants reported positive impacts of the pandemic, particularly partners working from home and being available to support (quote 28). They felt that their infant benefitted greatly from these alterations and having more interactions with the partner. A sense of relief that they were not ‘missing out’ on excitement of non-parents’ social life was also reported.

#### Interruption in health care services

In BC, over 70% of prenatal care and deliveries are provided by midwives and obstetricians, generally until 6 weeks postpartum.^24^ Thus, early postpartum period is often a transitional time from maternity care providers to general practitioners. According to our participants, throughout the pandemic, particularly during the first wave with temporary cease of non-urgent health care services, several participants reported being fell through cracks in this transition and not receiving the support they needed, particularly breastfeeding support and routine newborn check-ins. Some participants reported that their health care providers quit practice or did not provide in-person visits (quotes 29–30). This was particularly challenging for participants residing in rural and remote areas where there is often only one health care provider available. Participants reported feeling intense anxiety for not having access to postpartum and newborn health care services and that some health issues could be missed. Some participants reported delays in routine newborn and infant vaccination or difficulty in scheduling. A multiparous participant noted that she refrained from going to a public health clinic for infant’s routine vaccination due to the mask requirements and a lack of accommodation for her child with special needs. Another participant highlighted the hardships of delayed surgery for her infant. Frustration over limited number of visitors during prenatal care, delivery and baby’s check-in appointments were reported by several participants (quotes 31–32).

### Perceived postpartum care needs

Many participants expressed appreciation towards the health care providers and support that they received during the pandemic. Due to cancellation of some services particularly during the first wave of the pandemic, several participants expressed a need for maintaining health care services to have a sense of normalcy. The following needs were also identified:

#### In-person visits

Many participants particularly at the beginning of the pandemic reported being very cautious about taking baby to the clinic. These participants reported that virtual visits worked well for them and indeed they felt safer. In contrast, several participants noted that they needed in-person visits and that virtual care was not ‘enough’ (quote 33). They described virtual visits as distant and impersonal that was difficult to feel connected (quote 34). Particularly participants noted a need for in-person visits for infant’s check-in as they were concerned some health issues can be missed through virtual visits (quote 35). Some participants who experienced a blended visit with a pre-visit phone interview followed by a short in-person visit described these hybrid visits as being rushed and not providing sufficient time to connect with their care provider.

#### Allowing support persons

Several participants reported disappointments and anxiety for not having a support person during various medical appointments (quotes 36–37). Not allowing a support person was described as one of the most challenging consequences of the pandemic that participants had to deal with during their pregnancy and early motherhood.

#### Providing information/education/support groups

A great need for up-to-date information and education about the pandemic and updates on the latest adaptation in health care system in response to the pandemic was identified. Participants frequently reported frustrations about constant changes in guidelines and clinic protocols. Participants reported that they needed information on how to keep baby safe and what precautions are required in day-to-day life and clinic visits (quotes 38–39). Participants also noted a need for alternative and safe mother–baby groups to replace the cancelled in-person groups.

#### Mental health and social support

Many participants highlighted a need for their concerns and challenges to be validated and acknowledged by health care providers (quote 41). A need for formal mental health support in the form of counselling, support groups and meditation programmes was also noted (quote 40). Long waitlist and costs associated with mental health services were reported as barriers, and a need for no-cost/affordable mental health services was highlighted. Some participants noted that their health care providers were among the few people that they visited in-person and interacted with during the pandemic. Thus, they reported a great reliance on their health care providers for connections and support (quote 42).

#### Pro-active check-ins

Several participants reported that they would like their health care providers to actively reach out and check in (quote 43). They identified these pro-active check-ins as a source of psychological and social support.

## Discussion

The findings of this qualitative study provide insights on the experiences of postpartum people during the first year of the COVID-19 pandemic – from the initial surge in cases during the first wave to the peak of the third wave in BC, Canada. Our findings showed that the pandemic resulted in a plethora of unprecedented changes and experiences. Isolation and the closely concomitant experience of loss/lack of support were the two most dominant experiences observed in our data, particularly for immigrants and people without partners. Studies conducted in other regions also found loneliness and isolation to be the prominent pandemic-related experiences during the postpartum period.^25,26^

Anxiety and grief were two common psychological responses in our data. An area of concern for our participants was anxiety about the unavailability of reliable and up-to-date information regarding COVID-19 that has been also reported in the previous studies. Immediate lockdowns in various parts of the globe followed by instant shifts in the provision of health care and lack of up-to-date information created confusion and uncertainty for many perinatal people.^27,28^ The literature has highlighted an intense need among this population to understand the structure of provision of care in the new environment and how to safely access the pregnancy and postpartum services.^11,27^ A multi-national survey conducted in June 2020 showed that several months after the World Health Organization declaration of the global pandemic, perinatal people were still unsure and precarious about how they could best protect themselves and baby from the virus.^27^ Sixty percent of postpartum people in a Chinese study conducted between May and July 2020 reported a need for maternal and infant protection guidance.^29^

Restrictions on the number of support persons allowed to accompany the person on appointments were also commonly discussed as a source of disappointment. Not having support persons present during labour was also identified as a source of fear in postpartum people in the United Kingdom.^30^ A Canadian study of 57 postpartum people conducted between June 2020 and January 2021 found that limiting the number of support persons disproportionately affected those with health complications during and after delivery.^12^ In addition to providing psychological support, having support persons during labour and delivery has been linked to better pregnancy outcomes such as a shorter labour and fewer emergency caesarean and neonatal intensive care unit (NICU) admissions.^31^

The shift to virtual care garnered mixed feedback from our postpartum participants. While many appreciated the availability of virtual appointments, some participants described these visits impersonal and expressed concerns that health issues might be missed through a phone or video assessment. Similarly, in a qualitative study of postpartum experiences of social and health care support during the COVID-19 pandemic in the United Kingdom, participants perceived virtual health care as an ‘impersonal check box’ (p. 517) that was insufficient to address their health care needs.^32^ Karavadra et al.^30^ found similar findings in another UK study and reported that communicating health information during virtual visits was sometimes challenging due to a lack of privacy (isolating with others at home), feeling uncomfortable to disclose sensitive issue such as mental health struggles and technology issues with Internet connection. Others have also found that virtual health care might have limitations in patient-perceived confidentiality and level of care among non-perinatal populations.^33^ An unexpected finding of our study was the participants’ need for ‘pro-active’ check-ins. Several participants noted that they wanted to receive check-in calls from their health care providers even if they did not have a scheduled appointment. It is possible that given the isolation and public health restrictions, participants viewed their health care providers as a source of social support, as noted by some participants in our study.

Interruptions and delays in delivery of routine health care services, particularly breastfeeding, well-baby visits and childhood vaccination, were frequently reported, similar to another Canadian study.^12^ Concerns related to infant’s development were frequently noted with a focus on immune system development (due to excessive use of sanitizers, lack of exposures to common bacteria/viruses), psychosocial development, facial emotion recognition ability (due to face covering) and increased screen time and its consequences. While little research has been done on this topic, a previous research showed that children born in China during the severe acute respiratory syndrome (SARS) pandemic exhibited statistically significant delays in walking independently, saying a complete sentence, counting 0–10 and undressing him/herself for urination compared to those born after the pandemic.^34^ In a large longitudinal study of child neurodevelopment in the United States, Deoni et al. compared general childhood cognitive scores of children born during the COVID-19 pandemic to those born during 2011–2019. They found that the pandemic group had significantly lower verbal, motor and overall cognitive performance compared to children born pre-pandemic. They reported that the impact was more evident among males and children living in lower socioeconomic families.^35^

Policy and practice implications of our findings are several. Our results showed that virtual care, despite being convenient, was perceived inadequate by some postpartum people. During public health crises such as pandemics, a complete shift to virtual care may leave some postpartum people feeling anxious for not receiving the care that they or their infants need. Offering an in-person visits as well as allowing support persons in appointments for this population during pandemics is essential to ensure they receive adequate support and health care services.

Our findings also highlighted some misconceptions about the pandemic, particularly around the safety of COVID-19 vaccine for infants and while breastfeeding. Previous research also shows that postpartum people reported concerns about the safety of COVID-19 vaccine for baby while breastfeeding.^36^ A systematic review of 30 articles on the effects of COVID-19 vaccination (mostly mRNA vaccines) during lactation found that COVID-19 vaccine is safe for both breastfeeding person and the breastfed baby.^37^ It is important to provide up-to-date information as evidence indicates that most lactating people require more information to ensure the safety of the vaccine during breastfeeding and that they would receive the vaccine once they have access to reassuring evidence about vaccine safety.^36^

Concerns about child development were very evident in our findings. The preliminary evidence supports that the COVID-19 pandemic can negatively impact early child development, even in the absence of the infection. It is essential to monitor the children born during the pandemic, particularly those more vulnerable to developmental issues.^35^ Future longitudinal research to examine the long-term impact of pandemic on children’s physical and psychological development is also needed.

### Limitations

While collection of data immediately after the onset of pandemic throughout the first year and large sample size are strengths of this study, the study also has several limitations. This is a convenient sample, and most participants in this BC study were highly educated, White and had partners; thus, findings might not be applicable to populations with different demographics or regions with different COVID-19 outbreak intensities. The pandemic can intensify pre-existent health care inequalities during the postpartum period for marginalized people; hence, these populations may experience further impacts as the result of public health restrictions or health service interruptions related to the pandemic.^38^ The open-ended questions were constructed by the research team and were not piloted. We also did not collect data on participants’ COVID-19 status. It is possible that the experience of the people who contracted the virus could be different from those who did not.

## Conclusion

Responsive health care services (e.g. offering in-person visits for those who need, allowing support persons, providing up-to-date information and alternative venues for mother–infant groups to meet and connect, appreciating the potential role of primary care providers as a source of social and emotional support during the public health crises) are required to address the emerging and ongoing needs of postpartum people and improving postpartum care during the pandemic. Future studies are needed to explore mid-term and short-term impacts of pandemic on this population and early developmental outcomes.

## Declarations

**Ethics approval and consent to participate:** This study was reviewed and approved by the University of British Columbia Conjoint Health Research Ethics Board (approval id. H21-00489). All participants signed an informed consent form.

**Consent for publication:** Not applicable.

**Availability of data and materials:** Data are not publicly available.
